# Towards an overarching model for electronic medical-record systems, including problem-oriented, goal-oriented, and other approaches

**DOI:** 10.1080/13814788.2017.1374367

**Published:** 2017-11-17

**Authors:** Huibert Tange, Zsolt Nagykaldi, Jan De Maeseneer

**Affiliations:** a Department of Family Medicine, Maastricht University, Maastricht, The Netherlands;; b Department of Family & Preventive Medicine, University of Oklahoma, Oklahoma, OK, USA;; c Department of Family Medicine and Primary Healthcare, Ghent University, Ghent, Belgium

**Keywords:** Problem-oriented record, goal-oriented record, patient centredness, multimorbidity, primary care

## Abstract

There is no consensus among health professionals on how to structure medical records to serve clinical decision-making. Three approaches co-exist (source-oriented, problem-oriented, goal-oriented), each suiting a different subset of patients. In primary care, the problem-oriented approach is dominant, but for patients with multiple conditions (multimorbidity) the goal-oriented approach seems more appropriate. There is a need to combine different approaches in one medical-record system. In this article, we explain some misconceptions about ‘problems’ and ‘goals’ that hinder the way to consensus. When putting the approaches into historical perspective, it becomes evident that each relates to a different definition of health. Each approach has its specific merits that should be preserved even when health definitions change. Hence, we combine the merits of each approach into one overarching model, as to show the way to a new generation of electronic medical-record systems that can serve all patients. This model has three levels: a level of problems, diseases, and patient goals, a level of (shared) objectives, and a level of action plans and results.

Key messages

- Medical-record systems are aligned to either diseases, problems, or goals.
- There is a no consensus about which orientation is best. Each orientation reflects another definition of health.
- In primary care there is a need to combine different orientations. We present an overarching model for medical-record systems that combines them.

## Introduction

Medical record keeping is a continuous source of dispute. In a recent viewpoint, Martin and Sinsky express their frustration about current practice [1]. They argue that the medical record increasingly serves the purposes of bureaucrats and disregards the perspectives of patients and clinicians. This argument fits into the widespread belief among clinicians that they have lost some of their autonomy. However, the message that clinicians feel estranged from their information system tells more. It is also the expression of a lack of common view among clinicians of how a medical-record system should look like. We aim to provide such common view. We restrict this discourse to medical records, thus leaving out personal health records, which are part of another discourse.

For health professionals, the primary purpose of the medical record is to support clinical decision-making by providing them with readily accessible medical data and archiving these data for continuity of care. However, there is no consensus about how it should be structured to serve this purpose. Currently, there are three competing approaches: the source-oriented, problem-oriented, and goal-oriented model. In the 1990s, vivid discussions took place about which model is best [2–5]. Since the rise of electronic medical-record systems, these discussions ceased while sociotechnical issues became more urgent. Nevertheless the controversy remained.

Each model suits a subset of patients, but the trouble is that different models do not go together in one medical-record system, which makes it impractical, if not impossible, to serve all patients best. This is particularly the case for primary care with its growing share of patients with multiple health conditions. This notion recently elicited an academic discussion in our transatlantic team about the philosophy behind these models. The members of our team have their roots in different medical-record traditions but are united in their belief that convergence of approaches is needed to further the development of electronic medical-record systems in the desired direction. What started as a conversation about the pros and cons of each model grew into a critical reflection on misconceptions, shifting health paradigms, and merits to preserve, and ended up with a proposal to combine these models. After all, the promise of electronic medical-record keeping goes further than computerizing the documentation process. The challenge is to use the potential of computers to provide the professional decision maker with actionable information from whatever point of view, whether it is source-oriented, problem-oriented, or goal-oriented. This is because the electronic medical record should support the needs of the clinician, not the other way around.

## Ill-defined concepts

Problems and goals are ill defined. Their meanings given by the founders of the problem-oriented and goal-oriented medical records [5,6], have been forgotten or misinterpreted. There is a contrast of understanding: the problem-oriented model starts reasoning with a clinical problem and considers a goal as part of its solution, while the goal-oriented model starts reasoning with a patient goal and considers a problem as an obstacle to its attainment. This has led to much confusion, even within our team. To understand this confusion, we reconsidered the etymological roots of these concepts [7].

- The word ‘problem’ has been derived from ancient Greek *προβάλλω*, which means: ‘to throw or lay something in front of someone.’ Usually, it refers to a situation regarded as unwelcome or harmful. A medical problem was initially defined by Weed as ‘something of the patient that needs attention’ [6]. This could be anything disturbing the patient’s health: from a symptom or sign to a well-diagnosed disease, from a genetic risk factor to a socio-economical problem. This broad definition became the leading principle of primary care in the Netherlands, Belgium, and other European countries. In American primary care, however, the definition of ‘problem’ narrowed to the biomedical domain and its use was restricted mostly to a set of medical diagnoses, also called ‘problem lists.’
- The word ‘goal’ refers to the object of a person’s ambition or a desired result. Further interpretation depends on the role of the person and the reach of his/her ambition. In the problem-oriented model, a goal refers to a specifically desired achievement, which is part of the solution to a health problem (e.g. a differential diagnosis holds the goal to eliminate possible causes). In the goal-oriented model, however, a goal reflects a higher-level and usually longer-term personal ambition of a patient to give sense to his/her life (e.g. the wish to visit friends once a week).

To settle this confusion, we suggest sticking to the definitions that give name to each model and to avoid other interpretations of these words. Therefore, a *goal* remains the personal ambition of a patient and a *problem* remains a health condition that needs attention. We suggest other words for an obstacle in the care process (*barrier*) and for a desired achievement within the scope of a problem (*objective*).

## Shifting paradigms

From a historical perspective, the contrast between different medical-record models is part of a broader discourse of shifting health paradigms.

- The *source-oriented* medical record has been in use for more than a century [8]. It was a product of the classical biomedical model of healthcare, when diseases were the driving force and health was defined as ‘the state of being free of disease or injury.’ Another product of that era was the International Classification of Diseases (ICD) [9].
- In 1948, WHO redefined health as ‘a state of complete physical, mental and social well-being [10].’ This definition prepared the way to a new health paradigm: the biopsychosocial model [11]. The *problem-oriented* medical record was introduced aiming to align clinicians to ‘the whole patient,’ with all his biomedical, psychological, and social problems [6]. Health problems and risk factors became the driving force. A new codex was developed: the International Classification of Primary Care (ICPC) [12]. Under this paradigm, family medicine grew out to a mature academic discipline.
- Currently, there is a growing discomfort with the WHO definition, since complete well-being for all (and even for any) people seems unattainable. Its desire would only lead to the further medicalization of society. Instead of solving patient problems that impede well-being, it is often more productive to support patient capabilities that advance well-being. This practice is already standard in rehabilitation medicine and elderly care, domains where many patients have multiple health conditions (multimorbidity). There is also a suitable classification framework: the International Classification of Functions (ICF) [13]. Even a new definition of health has been suggested: ’the ability to adapt and self-manage [14].’ Resilience against distress becomes the new health paradigm. It is the driving force behind the *goal-oriented* medical record, helping the patient reach his/her personal longevity goals [2,15].

Each new health paradigm is an extension of the previous, representing a broader view of health and covering a larger group of patients. Each new medical-record model, however, is only focusing on the newly covered patients. No single model fits all patients. Hence, for primary care and other disciplines with a broad variety of patients, one needs to integrate all existing models in one medical-record system. This is not only to provide the opportunity to include the whole case-mix but also to switch within individual patient records whenever the situation arises.

## Merits of each model

The first challenge is to redirect the academic discourse from a controversy between alternative models to a synthesis of complementary models. To this end, we elucidate the most valuable principles of each model.

- The main merit of the *source-oriented* model is its two-dimensional ordering principle, which facilitates information retrieval. The first dimension is the source of clinical data: a physician part (with subdivisions medical history, physical examination, and progress notes), a laboratory part, a radiology part, and so on. The second dimension is time, useful for sorting and filtering clinical data [16].
- The *problem-oriented* model has two big merits. The first is a comprehensive view of health: each problem, not only medical, needs attention (while the source-oriented approach tends to focus on diagnosing and treating the most prominent disease). Its second merit is the SOAP-cycle (Subjective observations, objective observation, assessments and plans), which makes decisions subject to systematic evaluation: observations and plans are linked to the assessment of problems [6].
- The biggest merit of the *goal-oriented* model is the fact that it puts the patient in the centre, giving him or her a leading role in setting priorities. This facilitates shared decision-making [15]. Besides, it aligns clinical notes to the patient’s perspective, which is an advantage for modern patients who seek access to clinical notes via a patient portal, such as OpenNotes [17]. Another merit is the focus on patient abilities instead of disabilities, thus enforcing patient self-management [2]. A fourth merit is its esteem of the total constellation of causes rather than one cause at a time. This invites health professionals to consider the complex interactions of multimorbidity models in relation to the personal and social context of the patient and to construct one integrated, patient-individual, health strategy [5].

## Merging the merits

**Figure 1.**
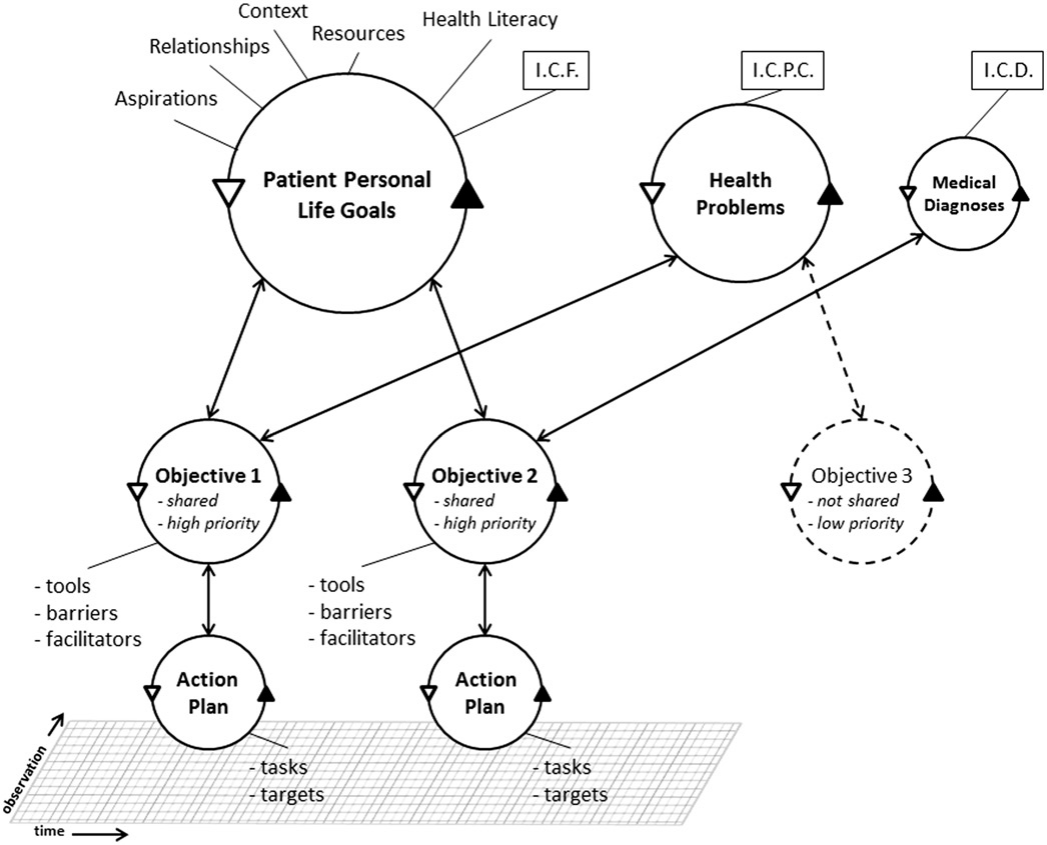
Revised model of goal-oriented care with the patient’s personal life goals in pole position. Arrows represent flows of information. ICD, ICPC, and ICF stand for the International Classifications of Diseases, Primary Care, and Functioning [9,12,13].

The second challenge is to combine these merits effectively in one overarching model, as a means to set the stage for electronic patient-record systems that accommodate the entire case mix of primary care. Figure 1 is the result of a pragmatic attempt.

The largest circle of Figure 1 is patient personal life goals, to emphasize that goals are the primary initiators of health objectives. Health problems and medical diagnoses are on the same level but smaller, to express their role as accommodators of health objectives. Health objectives that are rooted both in personal goals and problems or diagnoses are worked out by priority to action plans that result in new observations, which are organized in the medical record by source of observation and time. Goals, problems, objectives, and plans are presented as circles to convey that they are iteratively evaluated and adjusted. The connecting arrows between the circles are bidirectional, which implies that each goal, problem, objective, or plan can be re-evaluated when situations change. The action-plan circle is similar to the well-known SOAP circle, except that its assessment takes place in the context of a health objective instead of a health problem.

## Concluding remarks

To regain control of the medical record, clinicians should stop quarrelling about which model is best and find a solution that serves all current health paradigms. If a suitable solution is not established, electronic medical-record systems may degrade to real accountability tools and health professionals may eventually resort to the (patient-maintained) personal health record for clinical reasoning, which makes them totally out of control of their documentation.

We hope that this article will help clinicians speak one language and combine the best features of each model in one system. Our ideas have been inspired by experience from primary care, but may also be relevant for other disciplines. We encourage further discussion to improve the model, to find support among health professionals and patients, and to communicate the underlying philosophy to implementers of medical-record systems.

## References

[1] Martin SA, Sinsky CA. The map is not the territory: medical records and 21st century practice. Lancet. 2016;388:2053–2056. 27125861 10.1016/S0140-6736(16)00338-X

[2] De Maeseneer J, Boeckxstaens P. James Mackenzie Lecture 2011: multimorbidity, goal-oriented care, and equity. Br J Gen Pract. 2012;62:e522–e524. 22782000 10.3399/bjgp12X652553 PMC3381278

[3] Donnelly WJ, Hines E, Brauner DL. Why SOAP is bad for the medical record. Arch Intern Med. 1992;152:481–484. 1546910

[4] Feinstein AR. The problems of the problem-oriented medical record. Ann Intern Med. 1973;78:751–762. 4711779 10.7326/0003-4819-78-5-751

[5] Mold JW, Blake GH, Becker LA. Goal-oriented medical care. Fam Med. 1991;23:46–51. 2001782

[6] Weed LL. Medical records that guide and teach. N Engl J Med. 1968;278:593–600. 5637758 10.1056/NEJM196803142781105

[7] Stevenson A, editor. Oxford dictionary of English. Oxford: Oxford University Press; 2010.

[8] Reiser SJ. The clinical record in medicine. Part 2: reforming content and purpose. Ann Intern Med. 1991;114:980–985. 2024867 10.7326/0003-4819-114-11-980

[9] WHO. The ICD-10 classification of mental and behavioural disorders: clinical descriptions and diagnostic guidelines. Geneva: World Health Organization; 1992.

[10] WHO. WHO definition of health: World Health Organization; 1948 [cited 2016 September]. Available from: http://www.who.int/about/definition/en/print.html.

[11] Engel GL. The need for a new medical model: a challenge for biomedicine. Science. 1977;196:129–136. 847460 10.1126/science.847460

[12] World Organization of National Colleges A, and Academic Associations of General Practitioners/Family Physicians. ICPC-2: international classification of primary care. Oxford (UK): Oxford University Press; 1998.

[13] WHO. International classification of functioning disability and health: ICF. Geneva: World Health Organization; 2001.

[14] Huber M, Knottnerus JA, Green L, et al. How should we define health? BMJ. 2011;343:d4163. 21791490 10.1136/bmj.d4163

[15] Mold JW, Hamm R, Scheid D. Evidence-based medicine meets goal-directed health care. Fam Med. 2003;35:360–364. 12772939

[16] Tange HJ. How to approach the structuring of the medical record? Towards a model for flexible access to free text medical data. Int J Biomed Comput. 1996;42:27–34. 8880266 10.1016/0020-7101(96)01178-6

[17] Wolff JL, Darer JD, Berger A, et al. Inviting patients and care partners to read doctors’ notes: OpenNotes and shared access to electronic medical records. J Am Med Inform Assoc. 2017;24:e166–ee72. 27497795 10.1093/jamia/ocw108 PMC7651929

